# Informative missingness in electronic health record systems: the curse of knowing

**DOI:** 10.1186/s41512-020-00077-0

**Published:** 2020-07-02

**Authors:** Rolf H. H. Groenwold

**Affiliations:** 1 Department of Clinical Epidemiology, Leiden University Medical Centre, Leiden, the Netherlands; 2 Department of Biomedical Data Sciences, Leiden University Medical Centre, Leiden, the Netherlands

**Keywords:** Prediction modelling, Missing data, Routine care data

## Abstract

Electronic health records provide a potentially valuable data source of information for developing clinical prediction models. However, missing data are common in routinely collected health data and often missingness is informative. Informative missingness can be incorporated in a clinical prediction model, for example by including a separate category of a predictor variable that has missing values. The predictive performance of such a model depends on the transportability of the missing data mechanism, which may be compromised once the model is deployed in practice and the predictive value of certain variables becomes known. Using synthetic data, this phenomenon is explained and illustrated.

## Background

The amount of data that are currently being opened up for biomedical research are unprecedented [1]. Some argue that the sheer size of for instance electronic health records (EHR) datasets, in combination with its representativeness of daily clinical practice, carries an enormous potential for research that is relevant for clinical practice [2–5]. It provides ample opportunity to develop, e.g. clinical prediction models—predicting either diagnosis or prognosis—that may guide clinical decision making about treatment initiation or treatment switching [6].

However, missing data are common in routinely collected health data and often missingness is informative [7, 8]. This predictive information can be incorporated in a prediction model, for example by including an additional variable that indicates whether a predictor variable has missing values [9–11]. In what follows, it is illustrated that the predictive performance of such a model depends on the transportability of the missing data mechanism, which may be compromised once the model is implemented and the predictive value of variables becomes known.

## Informative missingness in electronic health records data

An example of a clinical prediction model is the Score model, predicting the probability of developing cardiovascular disease [12]. For such a model, high levels of certain biomarkers, for example high serum cholesterol levels, may indicate an increased risk of developing cardiovascular disease. Not only the actual values of a measured biomarker may carry information about the cardiovascular risk, also when the measurement was made, or how frequent measurements were made may be informative. In a recent study, it was found that for many commonly used laboratory measurements, the moment at which the test was requested was a better predictor of the risk of death within 3 years, than the actual result of the test [13].

Whether a measurement was made at all, may be informative too. Suppose we wish to develop a prediction model of the cardiovascular risk of patients who visit their general practitioner (GP). Likely, for a large proportion of patients no measurement of cholesterol is available in the electronic records of the GP [14], probably because the GP saw no need to measure it. Perhaps, at earlier consultations the cardiovascular risk was—implicitly or explicitly—considered too low to request a cholesterol measurement. In this situation, absence of a cholesterol measurement is in fact informative; one probably has a more favourable prognosis if the measurement is missing than if the measurement has been taken, irrespective of the measured value. We could incorporate this information in a cardiovascular risk prediction model; the lower the cholesterol the more favourable the prognosis and the lack of a cholesterol measurement is most favourable. When developing a clinical prediction model, an indicator for missingness could be added to a regression model [10, 15, 16]. Also, machine learning techniques such as classification and regression trees can accommodate missing data by including separate categories for missing values [17, 18]. General limitations of this approach have been described elsewhere [10].

## The curse of knowing

What is the predictive value of the aforementioned model that incorporates informative patterns of missing data? That is a question about transportability of a prediction model. Many factors are related to the transportability of a prediction model, including changes in patient characteristics (‘case mix’) [19], changes in administered treatments [20, 21] and changes in predictor measurement procedures [22]. Here, we focus on missing data and transportability.

Suppose we fast-forward time and the abovementioned cardiovascular risk model has been deployed in practice. If the considerations and reasons for taking a measurement were the same when developing the model as they will be at the time when the model is deployed in practice, then the presence or absence of a (e.g. cholesterol) measurement remains informative. But the moment a doctor knows that measuring cholesterol is informative, that knowledge may influences her considerations of whether or not to measure cholesterol. In that case, the predictive value of presence or absence of a measurement changes. A feedback loop arises, when informative patterns in the data influence measurement practices that subsequently change the information that is captured by particular (missing) data patterns. Knowing that a variable carries predictive information may alter the considerations to measure it, which subsequently may affect the predictive value of that variable [23].

## Illustrative example

To illustrate the impact of changing considerations to measure a predictor or not, sets of synthetic data were generated. These data were used to quantify the impact of differences in missing data mechanisms when applying a prediction model that was derived under informative missingness. These artificial data serve to illustrate a phenomenon; real-world data are likely much more complex and missing data mechanisms may be much more intricate.

### Methods

To generate and analyse sets of synthetic data, the statistical software package R was used [24].

First, a dataset representing 20000 subjects was generated that consisted of 2 uniformly (*U(0,1)*) distributed predictors; one predictor (*P*) was considered to be potentially observed, the other (U) was considered to be unobserved in all subjects. Also, for each subject, a binary outcome variable was sampled from a Bernoulli distribution, with probability dependent on both predictors, such that the outcome (*Y*) was present in approximately 34% of subjects: *P*(*Y* = 1|*P*, *U*) = 1/(1 + exp(−(−5 + 3*P* + 5*U*)))*.* Furthermore, the observed predictor was assumed missing in approximately 50% of subjects, where missingness (*R*) was dependent on both predictors: *P*(*R* = 1|*P*, *U*) = 1/(1 + exp(−(3−2*P*−2*U*−4*PU*))). Binary logistic regression analysis was applied to estimate a model predicting the outcome (dependent variable), based on the observed predictor *P* (independent variable). Four different approaches were implemented to handle missing data: (i) missing values of *P* were imputed with a value of zero and a variable indicating missingness was added to the model (‘zero imputation’); (ii) missing values of *P* were imputed with a mean of observed values of *P* and a variable indicating missingness was added to the model (‘mean imputation’ )[11]; (iii) analysis of only those subjects with an observed value of *P* (‘complete case analysis’); (iv) multiple imputation by chained equations was used to impute missing value of P (‘multiple imputation’). For the latter, the R package mice was used [25], with default settings, and observed values of *P* and the outcome (*Y*) were used for the imputation. A single imputed dataset was created, which was then analysed.

Next, a second dataset of 20000 subjects was generated, according to the same data generating mechanism as described above. Four scenarios of missing data mechanisms were applied. In the first scenario, the same missing data mechanism as described above was applied. In scenario 2, predictor *P* was measured in all subjects (i.e. no missing values). In scenario 3, predictor *P* was missing in a random 50% of subjects (i.e. uninformative missingness). In scenario 4, predictor *P* was missing in all subjects. For each of these scenarios, the developed prediction models were then applied to generate predictions of the probability of the outcome. It was assumed that missing data were handled in the same way, when developing the model and when applying the model. For example, if missing data were multiple imputed in the development data, multiple imputation was also applied in the application data. The predicted probabilities of the outcome were compared to the risk of developing the outcome based on the data generating mechanism (prediction error). Also, the predictive performance of the model was quantified by relating the predicted probability of the outcome to the observed outcome by means of the c-statistic [26], the Brier score [27] and calibration-in-the-large [28]. As a reference, a model was developed in the first dataset without missing values of *P* and then applied in the second dataset, again without missing values of *P*.

## Results

Table 1 summarises the predictive performance of the different approaches to handle missing data in the different scenarios. Interestingly, zero and mean imputation appear to perform better in scenario 1 than the reference model that was developed and tested using data without missing values. The reason for this is that in scenario 1, missingness itself was predictive of the outcome and because missingness was dependent on both *P* and *U*, missingness contains more information about the outcome than the single variable *P* in the reference model.

**Table 1:** Measures of predictive performance under different scenarios of missing data

Scenario–method	Mean prediction error (SD)	RMSPE	C-statistic	Brier score	Calibration-in-the-large
*Reference*					
No missing values	− 0.009 (0.244)	0.244	0.663	0.209	0.016
*Scenario 1*					
Zero imputation	− 0.004 (0.217)	0.217	0.699	0.197	0.019
Mean imputation	− 0.004 (0.217)	0.217	0.699	0.197	0.023
CCA	− 0.005 (0.244)	0.244	0.618	0.239	0.017
Multiple imputation	− 0.005 (0.269)	0.269	0.622	0.216	0.021
*Scenario 2*					
Zero imputation	0.104 (0.245)	0.266	0.663	0.220	− 0.467
Mean imputation	0.104 (0.245)	0.266	0.663	0.220	− 0.467
CCA	0.104 (0.245)	0.266	0.663	0.220	− 0.467
Multiple imputation	− 0.042 (0.246)	0.249	0.663	0.211	0.199
*Scenario 3*					
Zero imputation	− 0.024 (0.292)	0.293	0.541	0.234	0.119
Mean imputation	− 0.040 (0.299)	0.302	0.541	0.239	0.210
CCA	− 0.104 (0.245)	0.266	0.662	0.220	− 0.461
Multiple imputation	− 0.043 (0.264)	0.268	0.663	0.212	0.207
*Scenario 4*					
Zero imputation	− 0.151 (0.278)	0.316	0.500	0.248	0.782

*Abbreviations*: *CCA* complete case analysis, *SD* standard deviation, *RMPSE* root mean squared prediction error. See main text for a description of the scenarios and details about the methods

**Fig. 1:**
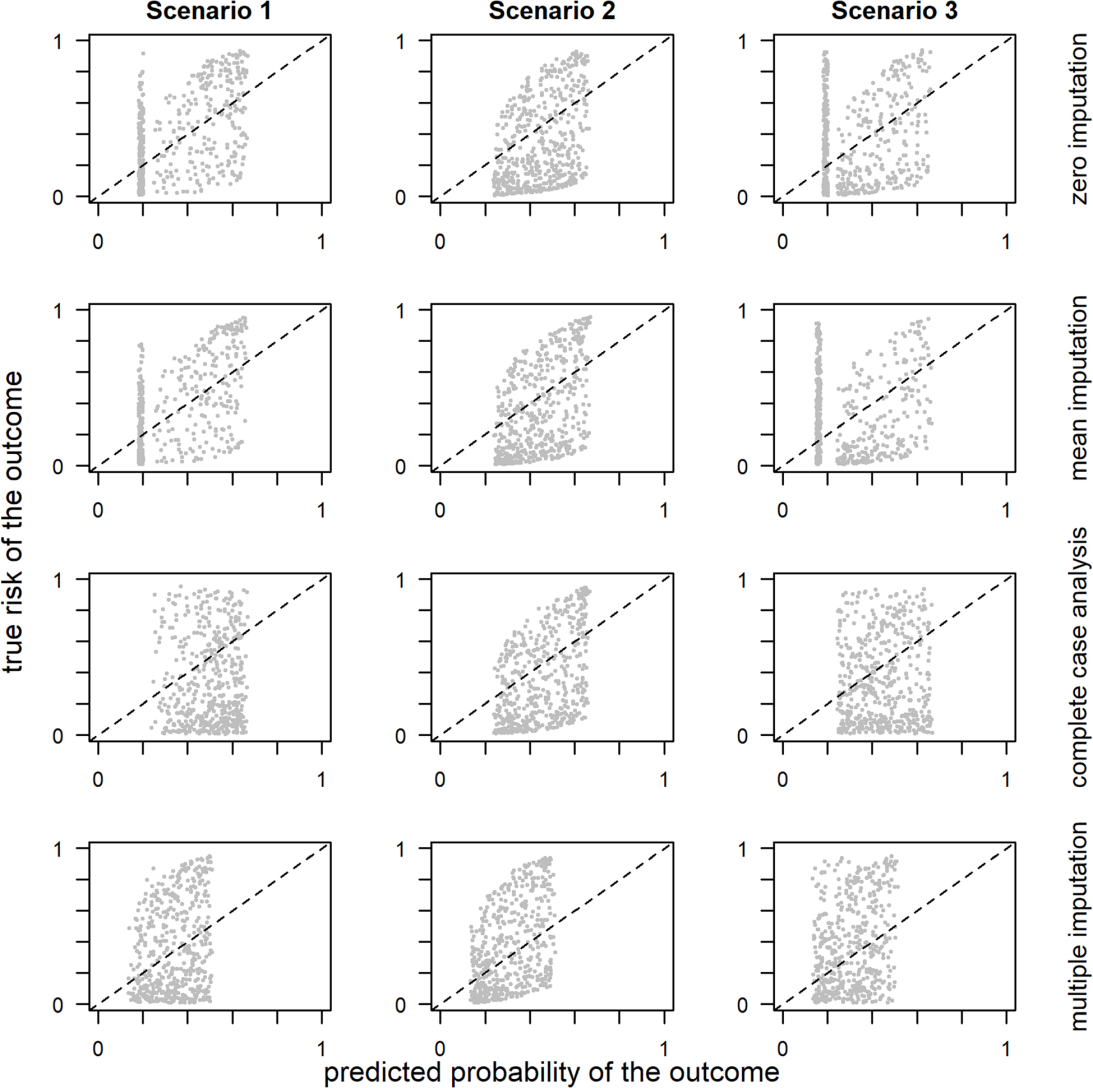
Impact of differences in missing data mechanisms when applying a prediction model that was derived under informative missingness using different approaches to handle missing data. Plots are based on 500 randomly sampled observations out of a dataset of 20000 observations. Predicted probabilities are jittered for visual clarity. See main text for a description of the scenarios and details about the methods

Figure 1 shows the impact of various missing data mechanisms on the predictive performance of the prediction model. The different approaches to handle missing data performed differently across the different scenarios. The left-hand panels are based on data with the same missing data mechanism as the data in which the prediction model was developed. For zero and mean imputation, the predicted probability of the outcome for subjects with a missing value of *P* equals the risk of the outcome amongst those with missing *P* values in the development data (approximately 0.2). This is not observed in the panels in the middle column (scenario 2), because there are no missing values in that scenario. In scenario 2, large calibration-in-the-large values indicates poor calibration of the model (Table 1). In scenario 3 (uninformative missingness), zero and mean imputation have poor performance, notably a poor c-statistic (Table 1). In scenario 4, only zero imputation can be applied, in which case the predicted probability of the outcome is the same for all subjects and therefore the c-statistic is 0.500.

## Conclusions

Informative missingness can be incorporated in a clinical prediction model, for example by including an additional variable that indicates whether a predictor variable has missing values. The illustrative example using synthetic data shows that the predictive performance of such a model depends on agreement between the missing data mechanism when developing the model and when deploying it in practice.

When developing a prediction model including one or more missing indicator variables, it is imperative to consider how the model will be used in practice. One aspect to consider is to what extent the doctor’s behaviour that gave rise to certain (informative) patterns in the data, such as the absence of a cholesterol measurement, is in fact transportable? For example, it might be expected that the model will be integrated in an electronic healthcare system, flagging high risk patients. In that case, healthcare professionals may remain ignorant of the particular input of the algorithm, in which case the missing data mechanism may remain similar to what it was when developing the model. However, when, e.g. a score chart is developed, it becomes explicit what the predictors are, in which case mechanisms of missing data likely change. Consequently, the predictive performance of the model likely will change too [23]. Instead of recommending a particular method to handle missing data in all situations, researchers who develop a prediction model should anticipate the missing data mechanism once the model is deployed in clinical practice.

The presented results are based on just one set of artificial data; by no means do they represent all possible scenarios of missing data and their impact on the performance of prediction models. However, although only a limited number of scenarios is considered, it illustrates the main point, namely, that relatively simple methods of dealing with (informative) missing data may have poor performance once the missing data mechanism changes. Importantly, none of the approaches performs best across all the different scenarios. If the missing data mechanism is informative and the same in the development data as in the data in which the model is applied, then the zero and mean imputation perform well (in these example data). However, if missingness has become a random process once the model is applied, multiple imputation appears to perform better. Rather than taking these observations as recommendations on how to handle missing data when developing a prediction model, the examples shows that choices about how to handle missing data should be guided by expectations about the missing data mechanism when the model will be deployed in practice. Future research is needed to quantify the impact of variations in missing data mechanisms on the transportability of prediction models.

In summary, commonly used methods to develop a prediction model can capture informative patterns of missing data in electronic health records data by including one or more missing indicator variables. When dealing with missing data in this way, it is paramount to anticipate how the prediction model will be used in practice and whether missing data mechanism are transportable to the setting of future application. Will a doctor’s actions and considerations stay the same once a prediction model is deployed in practice or will they change, e.g. based on characteristics of the model? If the latter is the case, the apparent informative patterns in electronic healthcare data may turn out to be uninformative once doctors start acting on them.

## Supplementary information

**Additional file 1.** R code.

## Data Availability

The datasets used and/or analysed during the current study are available from the corresponding author on reasonable request.

## Abbreviations

GP: General practitioner
